# Pyomo: Accidentally outrunning the bear

**DOI:** 10.1016/j.patter.2025.101311

**Published:** 2025-07-11

**Authors:** Miranda Mundt, William E. Hart, Emma S. Johnson, Bethany Nicholson, John D. Siirola

**Affiliations:** 1 Sandia National Laboratories, Albuquerque, NM, USA

**Keywords:** open-source, software, development, optimization, automation

## Abstract

Pyomo is an open-source optimization modeling software that has undergone significant evolution since its inception in 2008. Pyomo has evolved to enhance flexibility, solver integration, and community engagement. Modern collaborative tools for open-source software have facilitated the development of new Pyomo functionality and improved our development process through automated testing and performance-tracking pipelines. However, Pyomo faces challenges typical of research software, including resource limitations and knowledge retention. The Pyomo team’s commitment to better development practices and community engagement reflects a proactive approach to these issues. We describe Pyomo’s development journey, highlighting both successes and failures, in the hopes that other open-source research software packages may benefit from our experiences.

## The bigger picture

Research software forms the essential foundation of modern scientific discovery, particularly in data science, where computational tools translate raw data into meaningful insights. Optimization modeling, a mathematical approach that identifies optimal solutions amid constraints, represents one powerful application of such software, enabling better decision-making across numerous fields. The development practices used to create research software directly impact the quality, reliability, and reproducibility of scientific results across disciplines. As computational methods grow in complexity, the scientific community faces challenges in maintaining software that can adapt to evolving research needs while remaining usable and trustworthy. Open-source practices enable knowledge sharing and collaborative improvement, creating a pattern where better development approaches lead to more robust research software, which, in turn, produces more dependable scientific outcomes. The lessons learned from established software projects provide valuable guidelines for new and legacy projects alike, illustrating how strategic software development can enhance data-driven discovery across domains from healthcare to environmental science.

## Teaser

This perspective highlights the vital role of Pyomo in advancing scientific discovery through optimization modeling. It emphasizes how open-source practices improve software robustness, reproducibility, and community collaboration. The authors discuss Pyomo’s evolution since 2008, its development challenges, and lessons for future research software projects.

## Introduction

Mathematical modeling is a fundamental process in many aspects of scientific research, engineering, and business. The widespread availability of computing has made the numerical analysis and optimization of mathematical models commonplace activities.^1^ The Pyomo (https://www.pyomo.org) software package was developed to support new approaches to model and solve optimization problems.

Originally called the Coopr software library^2^ (no longer accessible), Pyomo gradually transformed into the open-source optimization tool that exists today. In its current form, Pyomo is accessible across all major operating systems (Linux, Windows, and MacOS) and all currently supported Python versions (3.9+) and averages 10,000 downloads from Pypi.org per day (per https://pypistats.org/packages/pyomo).

The probability that a research software project is still active after 14 years is less than 20%.^3^ The question is, how did Pyomo become what it is today? How did it grow from a small research software into a widely used open-source package that is the basis for multiple books^1^^,^^4^^,^^5^^,^^6^ and that supports numerous extensions for advanced modeling^7^^,^^8^^,^^9^^,^^10^^,^^11^ while surviving against the staggering odds for research software projects? The answer is: mostly by accident.

By fighting to stay ahead of each latest challenge, threat, or problem, we managed, in the end, to beat the odds and survive where other projects have failed. Internally, we have come to call this “outrunning the bear,” a reference to a common idiom of unknown origin in the United States that means, “you do not need to outrun the bear, you just need to run faster than the person next to you”—meaning you do not need to be perfect, but you do need to keep moving and outlast your competition.

In this perspective, we will explore how Pyomo changed over time, covering topics from the core tenets of our development philosophy to our motivations for changes in design and development pipelines as we adapted to meet the needs of our users and ourselves as researchers. We hope readers of this article can learn from some of our good (and bad) choices over the years and glean some helpful knowledge they can apply to their own open-source research software.

### A matter of (changing) principles

A matter of principle: a situation that requires something be done a certain way because one believes it is the only right way.^12^

When Coopr was created in 2008,^2^ Hart et al. laid the initial foundation for future development decisions. Their six main development principles were open-source licensing, customizable capabilities, solver integration, abstract models and concrete instances, flexible modeling, and portability. These tenets have persisted over time, and they have been augmented by several additional principles: managed approach to backward compatibility (API stability), targeted dependency management, and automation for development, productivity, and transparency.

These principles informed a variety of design and tool choices. For example, the need for flexibility and portability (plus an interest in emerging technologies) led to Python as the base language for Pyomo. While Python was by far the most popular and widely used language in 2024 (per the TIOBE index: https://www.tiobe.com/tiobe-index/), in 2008, the primary languages in use were Java, C, and C++. Python introduced new capabilities and modern functionality, including ease of use and intuitive object orientation, but the surrounding Python ecosystem was significantly smaller than it is today. This motivated the original Pyomo developers to co-develop the PyUtilib package (https://github.com/pyutilib/pyutilib), a collection of Python utilities intended to extend the base functionality of Python and its testing capabilities.

Managing two distinct projects with deep inter-dependencies, however, added strain to the already limited resources of the original development team. Over time, the functionalities written in PyUtilib became obsolete, as they were introduced directly into Python or other, better maintained Python packages. This experience inspired the tenet of “targeted dependency management.”^13^^,^^14^^,^^15^ Recognizing the challenges faced by the original developers, our current core team undertook a significant effort in the late 2010s to overhaul and minimize our dependencies. Today, Pyomo only has a single required dependency to run core functionality. Dependencies needed to run tests, build documentation, or exercise Pyomo’s more advanced and niche features are all set up to be optional. This allows us to manage both the stable and cutting-edge capabilities of Pyomo in a single Python package (and repository) while minimizing the installation overhead for users. This is an instance in which we accidentally outran the proverbial “bear.” While we made the changes primarily to reduce our own maintenance costs, recent literature shows that research software projects with fewer upstream dependencies are more likely to have long-term survival.^3^ Our willingness to change our principles over time has added protection against project abandonment.

**Tip**. Your goals will change over time. So, too, should your development choices. Don’t be afraid to reconsider old choices and try something new; otherwise, the bear may catch you.

### Scratching the (research) itch

Scratch the itch: to satisfy a desire or need for something.^16^

At its core, Pyomo is research software.^17^ It has been developed by researchers, for researchers, to achieve their research goals. This has not changed over the years—but Pyomo’s development process has. New research questions naturally drive the need for new capabilities. This research development cycle, however, does not map to the manner in which a production software, with many external users, is normally maintained. Pyomo’s development process evolved over the years to value backward compatibility for core functionality alongside a repository management strategy for distributing cutting-edge research capabilities without API stability guarantees. This value, in fact, is a direct result of conversations with the wider research community. When talking with potential early adopters of Pyomo, one of the authors had a conversation with a professor that still impacts how the Pyomo team manages backwards compatibility and breaking changes. The professor had been burned in the past by being an early adopter of a new research software package. Every time a new version of the software was released, his graduate students would lose months of precious research time trying to update their code to work with the latest version. The disruptions of his students’ productivity outweighed the benefit provided by the software, and he vowed to never make that mistake again. This story is now one of legend within the Pyomo development team, and it drives our development methodology and acts as yet another way in which we outrun the bear.

We have been able to balance the backwards compatibility concerns of Pyomo’s user community with our own research needs with the separation between what we refer to as “core” Pyomo offerings that promise long-term backwards compatibility versus “contributed” offerings that are still experimental and may change quickly with little notice. This balance between research-level and production-level software management has allowed Pyomo to emerge as a widely used tool for experimenting with and solving optimization problems across various applications, including energy,^18^^,^^19^ pharmaceuticals,^20^ chemical process design,^21^ critical infrastructure,^22^^,^^23^ and more. Research software faces a variety of unique challenges, including training,^24^ sustainable funding,^24^^,^^25^ and testing.^26^ These challenges have contributed to the rise of research software engineers,^27^ a professional designation that has grown significantly since its start in 2012. Partnered with this is the increase in the number of studies of the development of research software, including topics such as FAIR (findable, accessible, interoperable, and reusable) principles,^28^ community organization,^29^ and documentation.^30^

Pyomo developers have worked closely with the research software engineering community. Core developers attend community and technical meetings, and one of the core developers is a Steering Committee member for the US Research Software Engineer Association (https://us-rse.org). With this engagement comes a commitment to rigorous development practices in order to enable and accelerate high-quality, reproducible research results.

Our engagement with students is a clear example of this commitment. Because Pyomo is open source, accessible, and flexible, it is frequently used for graduate research that is then incorporated into Pyomo. However, we have long noted that “what is good for research is often not good enough for practice.”^31^ Thus, it has become standard protocol within Pyomo development to require software practices that improve quality (e.g., documentation, style guides, and testing) on all new functionality (with varying degrees of robustness). Specifics can be found in the Contribution Guide (https://pyomo.readthedocs.io/en/stable/contribution_guide.html). For example, developers outside of the core team are encouraged to incorporate new functionality into the contrib directory. This directory is regularly used to collaborate on new, experimental capabilities that are likely to need more research or substantial API changes. Conversely, source code within the core, common, and opt directories is promised a high level of stability, where substantial changes are conducted only for major version updates or as a result of significant defects or security fixes. When graduate research results in functionality that can be integrated back into Pyomo, we (or the advisor) work with the student to meet our minimum quality requirements.

This is, of course, not a perfect process. Much like industry software projects, we also suffer from low “bus factors”^32^; that is, the risk associated with the loss of core team members or, more ominously, “the number of team members who, if run over by a bus, would put the project in jeopardy.” This is common in research software due to the specialized and often interdisciplinary knowledge required to maintain complex, scientific code.^33^ For example, the Trust Region Framework (https://pyomo.readthedocs.io/en/stable/explanation/solvers/trustregion.html) was added to Pyomo in 2018, and it was originally developed during a graduate-level research project.^34^ Advancements on the original algorithm several years later^35^ required an update to the module. However, the graduate student was gone, and while the original source code had been reviewed (and there were tests), the knowledge on how it had been designed was lost. It had been caught by the bear. To update this algorithm, we ultimately had to start over from scratch and incorporate more documentation and stronger testing to protect our future selves.

**Tip**. Research is great, but remember: quality promotes longevity. Help yourself and future researchers by documenting your code, writing tests, and following better development practices. These will help keep the bear at bay.

### Fortune favors the bold (and restless)

Fortune favors the bold: English translation of the Latin proverb “audentes Fortuna iuvat,” a slogan used to emphasize the rewards of courage and bravery.^36^

One way to outrun the bear is to always keep running, especially with respect to development tools. The Pyomo development team has consistently aimed to incorporate modern technologies within our development and release processes. In particular, the developers have advocated for the use of tools and services that can promote productivity through automation and collaboration.^31^ We will explore a few examples from its history.

GitHub exploded in popularity in the mid-2010’s. Indeed, research trends in 2016 indicate that the topics of open-source software, git, and GitHub abounded.^37^^,^^38^^,^^39^^,^^40^ Pyomo, up to this point, had been leveraging subversion^41^ for version control, with the software hosted on a Sandia National Laboratories server. There were challenges with this approach, including collaboration limitations, more prescriptive development requirements, and performance implications. Though the transition from using svn to git and the relocation of the source code to GitHub would be disruptive,^42^ the Pyomo developers conducted the switch in 2015. This change opened up new opportunities for parallel development activities, local development practices, and the possibility of introducing continuous integration.^43^

Within 18 months of the move, core developers added the continuous integration tools AppVeyor (https://www.appveyor.com/, for Windows) and TravisCI (https://www.travis-ci.com/, for Linux) into the development pipeline. These services allowed the developers to test code as it was changed to quickly identify defects and prevent work-stopping breakages, which enabled them to focus on creating new capabilities and finding answers to pressing research questions. These also allowed for the evolution of the expected development workflow to require that new functionality be tested and that all tests are passed before new code can be merged.

This choice, however, introduced several bottlenecks—the bear was once again gaining on us. For example, in the case of AppVeyor, the test suites were run sequentially; that is, if the developers wanted to test 4 different versions of Python, and one suite took an average of 30 min, then they were waiting for 120 min to get results. Additionally, none of the services offered coverage for MacOS, so this “supported” platform was not regularly tested. In late 2019, GitHub released their own version of a continuous integration tool: GitHub Actions (https://github.com/features/actions). By this point, if two or three core developers were actively engaging with and committing to pull requests on GitHub, they could expect to wait 4–6 hours to receive testing results. The bear was getting too close—it was time for another switch. The core development team began exploring GitHub Actions in November 2019, ultimately fully migrating off Travis and AppVeyor by mid-2020 (for full details, see the report by Mundt et al.^44^). The change resulted in decentralized testing options, improved productivity, and reduced complexity and maintenance across services and added MacOS testing.

This also allowed Pyomo developers to pursue more advanced testing pipelines, including transparent and regularly updated performance benchmarks (https://pyomo.github.io/performance/), with automated notifications when a degradation occurs and the ability to port the primary testing framework from nose (https://nose.readthedocs.io/en/latest/, inactive since 2015) to pytest (https://docs.pytest.org/en/latest/, the *de facto* standard for Python testing in 2025). The constant exploration of new tools and the bravery to fully adopt them have allowed us to consistently improve our productivity and have opened new testing, tracking, and maintenance opportunities that we had previously never considered.

**Tip**. The best tool is the one you know, until that tool disrupts your ability to do work. Sometimes the investment in learning and shifting to a new tool or service is worthwhile. It can widen the gap between you and the bear.

### It takes a village (and their graduate students)

It takes a village: proverb of unknown origin that means that an entire community of people must provide for and interact positively with children; the full phrase is, “It takes a village to raise a child.”^45^

The prevalence of open-source software grew as the internet became more widely available in the 1990’s.^46^ Several large, well-known efforts led the way, including the GNU Project, Apache, and Linux. In recent years, the importance of open-source software development in research and academics has been touted loudly, citing open science as a way to expedite innovation,^47^ enable reproducibility,^48^ and ensure longevity and sustainability.^3^^,^^17^ In other words, the more people are involved, the harder it will be for the bear to catch them all.

Pyomo engaged in true open-source collaboration early in its history. One of the earliest methods of engagement came in the form of the Pyomo Forum (https://groups.google.com/g/pyomo-forum), a Google group created in 2010 as a mechanism for users to ask questions, provide feedback, and subscribe to updates on development activities and releases. When Pyomo moved to GitHub in 2015, users were encouraged to use the built-in Issue system to report their concerns, sometimes using discussions from the Pyomo Forum as the basis for new issues. As Pyomo grew in popularity, users naturally migrated to using StackExchange, on which the pyomo tag gained regular usage. There is a weekly “Dev Call” hosted for the core developers, but all are welcome to join, particularly those who wish to contribute to Pyomo. The instructions to receive an invitation are plainly listed in the README.

All of these different mechanisms for discussing Pyomo, from usage to defects to contribution, succeed due to the code developers’ engagement. Many on the development team, for example, are subscribed to get regular emails about questions on StackExchange that are tagged pyomo. Developers have regularly responded to questions posted on the Pyomo Forum. When an email is received to the Pyomo core developer list, users can expect a response within a few days.

As a result, collaborators around the globe, from academia to government to industry, have contributed to the development of Pyomo. The existence of several solver integrations is a direct effect of this. One of the newest solvers to be supported is SAS Optimization (https://www.sas.com/en_us/software/optimization.html), a commercial optimization solver available from the SAS Institute. A developer from SAS created the interface and regularly attended the weekly Dev Calls to receive support, help, and updates to enable the functionality to be incorporated. Similarly, Pyomo developers regularly connect with the HiGHS^49^ development team to ensure continued and sustainable support. Pyomo has also worked with other commercial solvers, such as Gurobi (https://fico-xpress-optimization), and MOSEK (https://www.mosek.com) to receive licensing support to ensure that models, large and small, will remain compatible.

In addition, Pyomo developers directly act as liaisons within the wider optimization modeling community (with an emphasis on energy applications). For example, several core developers are shared across Pyomo, the Institute for the Design of Advanced Energy Systems (IDAES),^21^ and WaterTAP.^50^ These developers attend both Pyomo-specific team meetings as well as their collaborators’ meetings. They also strategically stagger software release dates and run cross-software test suites to guarantee compatibility (incidentally, the research of Malviya Thakur et al. into the survival rates of open-source research software projects found that those with downstream dependents were more likely to survive for longer^3^). Finally, they collaborate on funding proposals to ensure continued financial support for multiple parts of the ecosystem for long-term development, sustainability, and maintenance. While it may seem clear that engaging with our community is essential, we have found that these interactions have been instrumental in effectively repelling the bear and ensuring the continued growth and resilience of Pyomo.

The Pyomo development team, however, has its own limitations. No member of the team works full time on Pyomo. All of us have other projects and responsibilities that split our time. As a result, we face challenges addressing reported issues and reviewing new pull requests with as much expediency as our users and external collaborators would like. Additionally, because most funding supporting Pyomo is focused on research concerns, the team has to strategically prioritize development and support activities, which may not involve regular maintenance or buydown of technical debt.^51^ These struggles are shared across research software, where time fragmentation is a common complaint.^52^ We attempt to balance this by writing software maintenance activities as line items into funding proposals, frequently citing that critical infrastructure only remains reliable if the software that underpins it does as well.

The core message in this section is the importance of open-source collaboration in ensuring the longevity of a research software project. Much like a bear can take items from an empty campsite with ease, a research software project with little to no engagement is the most at risk for abandonment.

**Tip**. A group of people will always have more knowledge than a single person. The opensource software community has a lot to offer if you are willing to engage with it. An active group of people will intimidate the bear.

## Conclusion

Research into the survival of open-source research software projects is in its infancy. The paper by Malviya Thakur et al. is, in fact, the first of its kind to focus on survival rates for research software.^3^ While modern-day projects are able to build on the successes and mistakes of their predecessors, projects like Pyomo had very little data and experience upon which to build.

From its inception in 2008 to its current status as a pivotal tool in optimization research, Pyomo has balanced innovation, community engagement, and normal productivity challenges. While many of the foundational motivations established by Hart et al.^4^ have continued to guide its development, new practices, technologies, and challenges have catalyzed the evolution of Pyomo in new directions. This willingness to change led to the adoption of collaborative tools (like GitHub) and a robust testing infrastructure that spans all supported operating systems and Python versions.

These changes involved many challenges that are common concerns for modern research software. As the complexity and user base of Pyomo grew, so too did its issues, such as limited resources, knowledge retention, and sustainable funding streams. The core development team’s commitment to better development practices has eased these struggles by proactively adapting and regularly searching for opportunities for improvement. This has also led to a helpful spirit within the wider Pyomo community, creating opportunities for collaboration with industry, academia, and government.

True research software ecosystem sustainability requires a balance of research ambitions with software maintenance and community engagement. As the development of open-source research software continues to grow, the lessons learned from Pyomo’s development journey can serve as a valuable guide for other research software projects.

Our success can be attributed to a combination of backward compatibility, adherence to baseline coding standards, and the guidance of a dedicated team that helps us adapt to changing times. By engaging broadly with communities at the forefront of research, we have not only kept pace with emerging needs but have often been ahead of the curve in terms of capabilities and support. In this way, we have effectively outrun the bear, ensuring that Pyomo remains a relevant and powerful tool in the optimization landscape.

## Acknowledgments

Sandia National Laboratories is a multimission laboratory managed and operated by National Technology & Engineering Solutions of Sandia, LLC, a wholly owned subsidiary of Honeywell International Inc., for the US Department of Energy’s National Nuclear Security Administration under contract DE-NA0003525.

## Author contributions

Conceptualization, M.M., W.E.H., E.S.J., B.N., and J.D.S.; investigation, M.M.; writing – original draft, M.M.; writing – review & editing, M.M., W.E.H., E.S.J., B.N., and J.D.S.; funding acquisition, B.N. and J.D.S.; supervision, W.E.H., B.N., and J.D.S.

## Declaration of interests

The authors declare no competing interests.

## Declaration of generative AI and AI-assisted technologies in the writing process

During the preparation of this work, the authors used an on-premise generative AI tool in order to correct phrasing and tone for a subset of sections in addition to finalizing the language in The bigger picture. After using this tool or service, the authors reviewed and edited the content as needed and take full responsibility for the content of the publication.
